# Dynamically Tuning the Up-conversion Luminescence of Er^3+^/Yb^3+^ Co-doped Sodium Niobate Nano-crystals through Magnetic Field

**DOI:** 10.1038/srep31327

**Published:** 2016-08-09

**Authors:** Quanlan Xiao, Yuanhao Zhang, Han Zhang, Guoping Dong, Junbo Han, Jianrong Qiu

**Affiliations:** 1 Shenzhen Key Laboratory of Two-dimensional Materials and Devices (SKTMD), SZU-NUS Collaborative Innovation Center for Optoelectronic Science and Technology, and Key Laboratory of Optoelectronic Devices and Systems of Ministry of Education and Guangdong Province, Shenzhen University, Shenzhen 518060, P.R. China; 2 State Key Laboratory of Luminescent Materials and Devices and Institute of Optical Communication Materials, South China University of Technology, Guangzhou 510640, P.R. China; 3 Wuhan National High Magnetic Field Center and School of Physics, Huazhong University of Science and Technology, Wuhan 430074, P.R. China

## Abstract

In this work, we show here that the up-conversion luminescence of NaNbO_3_:Er^3+^/Yb^3+^ nano-materials can be modulated by magnetic field and a enhancement of up-conversion intensities by a factor of about 2 for Er^3+^:^*4*^*S*_*3/2*_ *→* ^*4*^*I*_*15/2*_ obtained at 30 T and about 5.4 for Er^3+^:^*4*^*F*_*9/2*_ *→* ^*4*^*I*_*15/2*_ obtained at 20 T. The increased up-conversion luminescence are mainly interpreted in terms of the enhanced non-radiation transition from ^*4*^*I*_*11/2*_ to ^*4*^*I*_*13/2*_ of Er^3+^ ions and the spin-orbital coupling (that is “mixing” effect) in crystal field by an external magnetic field. Meanwhile, we observed continuously spectra broadening with growing the magnetic field intensity, which is ascribed to the “mixing” effect induced by magnetic field and the difference of *g* factor of sub-bands. This bi-functional material with controllable optical-magnetic interactions has various potential applications, such as optical detection of magnetic field, *etc*.

The bi-functional magnetic-optical materials have received growing attention for their potential applications in high accuracy communication, magnetic resonance imaging (MRI), drug targeting or carrier, aircraft guidance and optical detection of magnetic field, due to their ability to be detected at different models, optically and magnetically^1^,^2^,^3^,^4^,^5^,^6^,^7^. Due to conventional magnetic-optical bi-functional materials are produced by coupling optical materials with magnetic ones, making the optical and magnetic phases separation, it is very difficult to realize an interaction between the optical and magnetic properties (e.g., tuning the optical properties using a magnetic field)^8^,^9^,^10^,^11^. Rare-earth (RE) ions doped materials are ideal candidates to realize these functions because they possess rich 4*f* energy levels in optical frequency, the tunability of magnetic-optical interaction by external magnetic field, and excellent photostability^12^,^13^,^14^,^15^.

Several recent works show that magnetic-optical interactions can be simultaneously observed in various materials by RE doping, for example, Er^3+^-doped nano-glass-ceramics^8^, YVO_4_:Er^3+^ 16,17, Gd_2_O_3_:Er^3+^,Yb^3+^ 11,18, NaGdF_4_:Er^3+^,Yb^3+^ 19,20, Nd^3+^ co-doped NaGdF_4_:Er^3+^,Yb^3+^ nano-crystals^21^, and so on. In this materials, the luminescence intensities of emission bands of RE ions are a gradual decrease (or increase) with increasing (or decreasing) applied magnetic field. In addition, in comparison to those substrates, NaNbO_3_ has Perovskite structure and excellent properties such as piezoelectric, ferroelectric, optoelectronics, nonlinear optical, and so on. In addition, we have studied the optical second harmonic generation (SHG) of single NaNbO_3_ micro/nano-crystals with various morphologies and sizes, which exhibited strong nonlinear SHG responses^22^. Considering its advantages and based on our research foundation, we chose NaNbO_3_ as the host material to dope with the classic up-conversion ionic pair, Er^3+^/Yb^3+^, which can convert low-energy infrared photons to high-energy visible light.

In this work, the effect of external magnetic fields on the magneto-upconversion luminescence (MUL) properties of emission bands of Er^3+^:^*4*^*S*_*3/2*_ *→* ^*4*^*I*_*15/2*_ and ^*4*^*F*_*9/2*_ *→* ^*4*^*I*_*15/2*_ transitions is studied, which results are quite different from the recent reports^8^,^11^,^16^,^17^,^18^,^19^,^20^,^21^. For instance, Moshchalkov *et al*. first showed the tuning of the luminescence of Er^3+^ doped nano-particles^16^,^17^, which up-conversion luminescence intensities are always suppressed by magnetic field, possibly due to the enhanced cross-relaxation process, reduced absorption cross, and improved local site symmetry. While in the present, the integrated luminescent intensity of Er^3+^:^*4*^*S*_*3/2*_ *→* ^*4*^*I*_*15/2*_ could increase to approximately 200% of the original value in the applied magnetic field if reached up to 30 T, and the integrated luminescent intensity of Er^3+^:^*4*^*F*_*9/2*_ *→* ^*4*^*I*_*15/2*_ could increase to approximately 540% of the original value in the applied magnetic field if reached up to 20 T. These results are mainly interpreted in terms of the enhanced non-radiation transition from ^*4*^*I*_*11/2*_ to ^*4*^*I*_*13/2*_ of Er^3+^ ions and the spin-orbital coupling (that is “mixing” effect) in crystal field by an external magnetic field. This remarkable tunability indicates that the studied nano-material can serve as a good optical-magnetic bi-functional material for various potential applications.

## Results and Discussion

### Phase purity, structure and morphology

**Figure 1:**
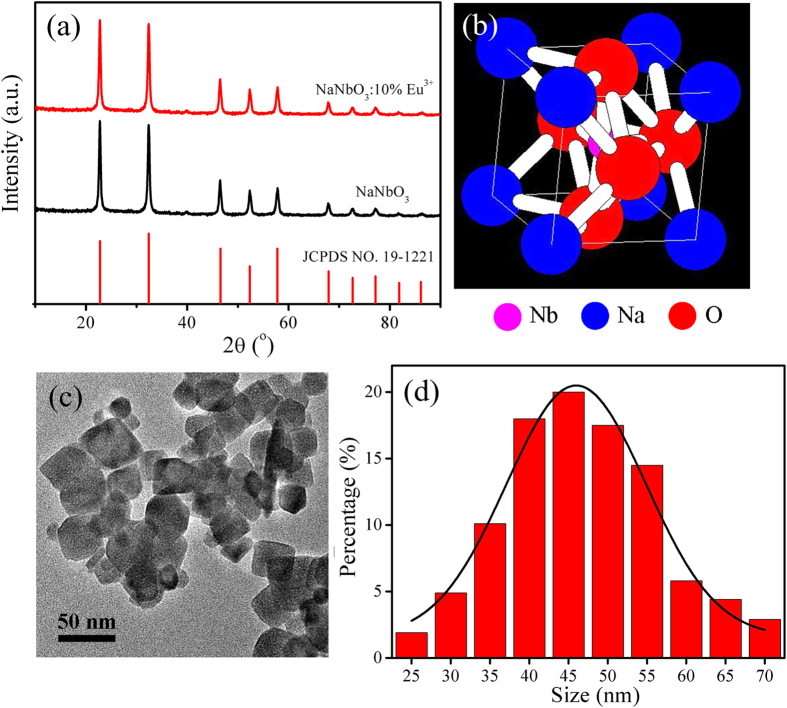
(**a**) XRD patterns of NaNbO_3_ and NaNbO_3_:2%Er^3+^,20%Yb^3+^ nano-crystals. (**b**) Crystal structure of cubic NaNbO_3_. (**c**) TEM image of NaNbO_3_ nano-crystals. (d) The size distribution of NaNbO_3_ nano-crystals.

High quality NaNbO_3_ nano-crystals were prepared by the Pechini sol-gel method. The XRD patterns and TEM image of as-prepared samples are shown in Fig. 1. From Fig. 1(a), we can confirm that all the as-prepared samples have cubic crystal structures, which are consistent with Joint Committee on Powder Diffraction Standards (JCPDS) Card No. 19–1221 (space group: *Pm-3m*(221), *a* = *b* = *c* = 0.391 nm), the interrelated crystalline structure is shown in Fig. 1(b). By co-doped Er^3+^/Yb^3+^ ions, no precipitating phase could be detected in the XRD patterns, demonstrating that there is no obvious influence on the phase when doping with Er^3+^/Yb^3+^ ions in NaNbO_3_ nano-crystals. As we may know, the ionic radii of Nb^5+^, Na^+^, Er^3+^ and Yb^3+^ cations are 64, 102, 89 and 87 pm, respectively. When Er^3+^/Yb^3+^ ions substitute the site of Na^+^ or Nb^5+^ ions, the values of mismatching ratio of ionic radius are 0.13 and 0.36, respectively. And the ionic radii of Er^3+^/Yb^3+^ ions is smaller than Na^+^ ions and larger than Nb^5+^ ions. It could be inferred that Er^3+^/Yb^3+^ ions are more likely to substitute the sites of Na^+^ ions than Nb^5+^ ions. Besides, NaNbO_3_ is a cubic crystal as shown in Fig. 1(b), Nb^5+^ ions occupy the body-centered and Na^+^ ions occupy the eight vertexes of the cube. Therefore, it is believed that Er^3+^/Yb^3+^ ions prefer to occupy the Na^+^ sites. Moreover, the TEM image and size distribution of as-prepared NaNbO_3_ nano-crystals are given in Fig. 1(c,d), it can be seen that the as-prepared NaNbO_3_ nano-crystals exhibit relatively uniform, well-dispersed nano-particles with narrow size distribution centered around 50 nm.

### MUL properties of Er^3+^/Yb^3+^ co-doped NaNbO_3_ nano-crystals

**Figure 2:**
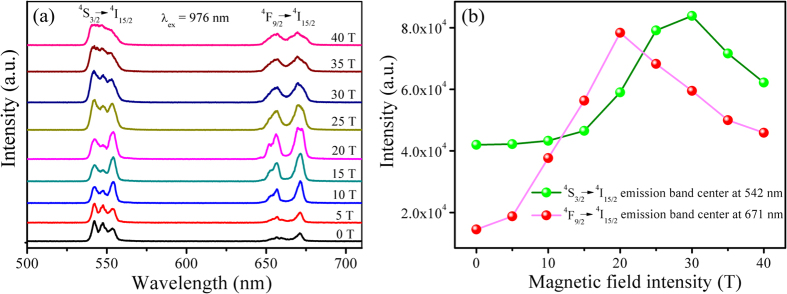
Effect of external pulsed magnetic fields (up to 40 T) on the MUL properties of the ^*4*^*S*_*3/2*_ *→* ^*4*^*I*_*15/2*_ and ^*4*^*F*_*9/2*_ *→* ^*4*^*I*_*15/2*_ transitions of the Er^3+^ ions (excited by 976 nm laser) in Er^3+^/Yb^3+^ co-doped NaNbO_3_ nano-crystals at 77 K [(**a**) emission spectra, (**b**) integrated luminescent intensities].

Figure 2 shows the effect of increasing the external magnetic field (up to 40 T) on the MUL properties of Er^3+^:^*4*^*S*_*3/2*_ *→* ^*4*^*I*_*15/2*_ and ^*4*^*F*_*9/2*_ *→* ^*4*^*I*_*15/2*_ transitions in NaNbO_3_:2%Er^3+^,20%Yb^3+^ nano-crystals at 77 K [(a) emission spectra, (b) integrated luminescent intensities]. The magnetic field is in a direction parallel to the exciting radiation. The emitted radiation is monitored in the direction parallel to the applied magnetic field as well as the exciting radiation. In the absence of the magnetic field, this sample emits very strong radiation with emission bands of 530–570 nm and 642–685 nm under 976 nm laser excitation (~150 mW power), corresponding to ^*4*^*S*_*3/2*_ *→* ^*4*^*I*_*15/2*_ and ^*4*^*F*_*9/2*_ *→* ^*4*^*I*_*15/2*_ transitions of the Er^3+^ ions, respectively^16^,^17^,^21^. Yb^3+^ ions act as the sensitizer for Er^3+^ ions. The integrated luminescent intensity of both emission bands first increases with the applied magnetic fields and then decreases with the applied magnetic fields increasing further. The integrated luminescent intensity of Er^3+^:^*4*^*S*_*3/2*_ *→* ^*4*^*I*_*15/2*_ could increase to approximate 200% of the original value in the applied magnetic field reached up to 30 T, and the integrated luminescent intensity of Er^3+^:^*4*^*F*_*9/2*_ *→* ^*4*^*I*_*15/2*_ could increase to approximate 540% of the original value in the applied magnetic field reached up to 20 T. It is evident from Fig. 2 that the emissions from the 4*f*^* n*^ shell electronic transitions (^*4*^*S*_*3/2*_ *→* ^*4*^*I*_*15/2*_ and ^*4*^*F*_*9/2*_ *→* ^*4*^*I*_*15/2*_) of Er^3+^ ions can be efficiently tuned by changing the applied magnetic field at 77 K.

In the present work, the effect of external magnetic fields on the MUL properties of emission bands of Er^3+^:^*4*^*S*_*3/2*_ *→* ^*4*^*I*_*15/2*_ and ^*4*^*F*_*9/2*_ *→* ^*4*^*I*_*15/2*_ transitions is quite different from the recent reports^8^,^11^,^16^,^17^,^18^,^19^,^20^,^21^, The main reasons for this result are interpreted as follows: on the one hand, the enhanced non-radiation transition from ^*4*^*I*_*11/2*_ to ^*4*^*I*_*13/2*_ of Er^3+^ ions, on the other hand, the combined with “mixing” effect (spin-orbital coupling, Hamiltonian model^23^,^24^) and Kramers’ degeneracy of Stark sub-levels in crystal field by an external magnetic field. The properties of transitions within the 4*f*^n^ configurations of lanthanide ions are strongly dependent on the environment of the ion in terms of differences in ion size or the ionic dependence of site distortions. Although Er^3+^ ions are in sites with cubic symmetry, the actual site symmetry of Er^3+^ ions in excited-states of ^*4*^*S*_*3/2*_ and ^*4*^*F*_*9/2*_ energy levels may be slightly lower due to the charge compensation and differences in ionic radius of Na^+^ (r = 102 pm) or Er^3+^ (r = 89 pm) ions. This lowering of the site symmetry results in crystal field splitting (Stark sub-levels) of the ^*4*^*S*_*3/2*_ and ^*4*^*F*_*9/2*_ energy levels. As we know, the Zeeman splitting behavior is complex. From Fig. 2, we can find that the number of splitting peaks of both ^*4*^*S*_*3/2*_ and ^*4*^*F*_*9/2*_ energy levels can’t relax the selection rules when applied magnetic fields, and there is no change of the number of splitting peaks applying magnetic field or not. Therefore, it could be suggesting that the Zeeman splitting effect may not be considered.

**Figure 3:**
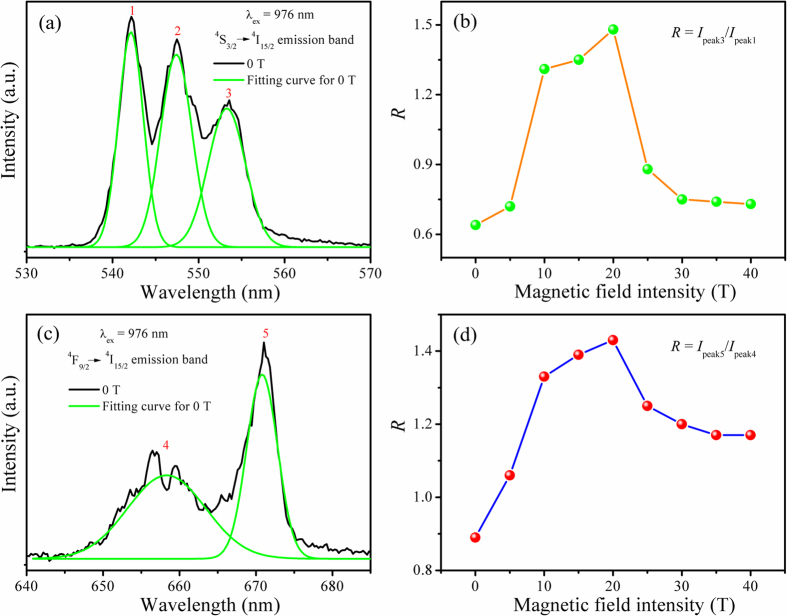
Emission spectra of Er^3+^:^*4*^*S*_*3/2*_ *→* ^*4*^*I*_*15/2*_ and ^*4*^*F*_*9/2*_ *→* ^*4*^*I*_*15/2*_ transitions in NaNbO_3_:Er^3+^,Yb^3+^ nano-crystals at zero magnetic field and the corresponding multi-peak Gaussian fitting curves, and the indexes *R* values of *I*(553 nm, peak3)/*I*(542 nm, peak1) and *I*(671 nm, peak5)/*I*(658 nm, peak4) at different magnetic fields at 77 K.

The “mixing” effect is the mixing of states by magnetic field. This effect exists only in condensed substances, in which the atomic states are split by the crystal field. Furthermore, in order to study the “mixing” effect on Stark sub-levels of the Er^3+^:^*4*^*S*_*3/2*_ *→* ^*4*^*I*_*15/2*_ and ^*4*^*F*_*9/2*_ *→* ^*4*^*I*_*15/2*_ transitions in magnetic field, the peaks of emission bands are fitted by the multi-peak Gaussian fit and are analyzed. The emission band of Er^3+^:^*4*^*S*_*3/2*_ *→* ^*4*^*I*_*15/2*_ is fitted for three peaks as 542 nm, 547 nm and 553 nm and the emission band of Er^3+^:^*4*^*F*_*9/2*_ *→* ^*4*^*I*_*15/2*_ is fitted for two peaks as 658 nm and 671 nm. We define the rates of the integrated intensities of the peaks at 553 nm and 542 nm of ^*4*^*S*_*3/2*_ *→* ^*4*^*I*_*15/2*_ transition and the peaks at 671 nm and 658 nm of ^*4*^*F*_*9/2*_ *→* ^*4*^*I*_*15/2*_ transition, those are, the indexes *R* = *I*(553 nm, peak3)/*I*(542 nm, peak1) and *R* = *I*(671 nm, peak5)/*I*(658 nm, peak4), which could be used to analyze the “mixing” effect on Stark sub-levels of the Er^3+^:^*4*^*S*_*3/2*_ *→* ^*4*^*I*_*15/2*_ and ^*4*^*F*_*9/2*_ *→* ^*4*^*I*_*15/2*_ transitions in different magnetic fields, as shown in Fig. 3. The “mixing” effect (spin-orbital coupling interaction) could cause an external energy (Δ*E*) to each non-degenerate energy level in applied magnetic fields, which can be described by the following equation (the related derivation shows in Supplementary)^25^:


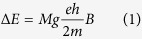


in which, *M* is magnetic quantum number, *g* is Lande factor, *e* is the electric charge, *h* is Planck’s constant, *m* is the mass of an electron, *B* is the magnetic field intensity. The equation (1) indicates the energy gap between the non-degenerate levels becomes larger with the increase of applied magnetic field, it is beneficial for the transition of the lower non-degenerate level and reduces non-radiative relaxation as a result of increasing the integrated luminescent intensity, which are consistent with the results of the experiment. In relatively weak magnetic field, the integrated luminescent intensities of both the emission bands of Er^3+^:^*4*^*S*_*3/2*_ *→* ^*4*^*I*_*15/2*_ and ^*4*^*F*_*9/2*_ *→* ^*4*^*I*_*15/2*_ transitions (Fig. 2(b)) and the indexes *R* [*R* = *I*(553 nm, peak3)/*I*(542 nm, peak1), *R* = *I*(671 nm, peak5)/*I*(658 nm, peak4)] values (Fig. 3(b,d)) increase with the applied magnetic fields increasing, this is because the spin-orbit coupling interaction plays a dominant role comparing with interactions between spin (orbit) and magnetic field. In relatively strong magnetic field, the interactions between spin (orbit) and magnetic field are more than the interaction of spin-orbit coupling making spin-orbit coupling interaction vanished, so the integrated luminescent intensities of both the emission bands of Er^3+^:^*4*^*S*_*3/2*_ *→* ^*4*^*I*_*15/2*_ and ^*4*^*F*_*9/2*_ *→* ^*4*^*I*_*15/2*_ transitions and the indexes *R* values decrease with further increasing the applied magnetic field. However, due the external energy Δ*E* of non-degenerate energy levels of ^*4*^*S*_*3/2*_ *→* ^*4*^*I*_*15/2*_ transition is stronger, the interaction of spin-orbit coupling is larger, there is a delay of the “mixing” effect for this transition as a result of the integrated luminescent intensity of the emission band of ^*4*^*S*_*3/2*_ *→* ^*4*^*I*_*15/2*_ transition begins to drop at 30 T, instead of 20 T for ^*4*^*F*_*9/2*_ *→* ^*4*^*I*_*15/2*_ transition. Er^3+^ ion has an odd number of 4*f* electrons (4*f *11), and shows therefore Kramers’ degeneracy in any crystal field. Magnetic field lifts all crystal field degeneracy. Therefore, in strong magnetic field, the sub-levels of both the emission bands of Er^3+^:^*4*^*S*_*3/2*_ *→* ^*4*^*I*_*15/2*_ and ^*4*^*F*_*9/2*_ *→* ^*4*^*I*_*15/2*_ transitions occur degeneracy, and the degeneracy increases with the applied magnetic fields increasing, as shown in Fig. 2(a).

**Figure 4:**
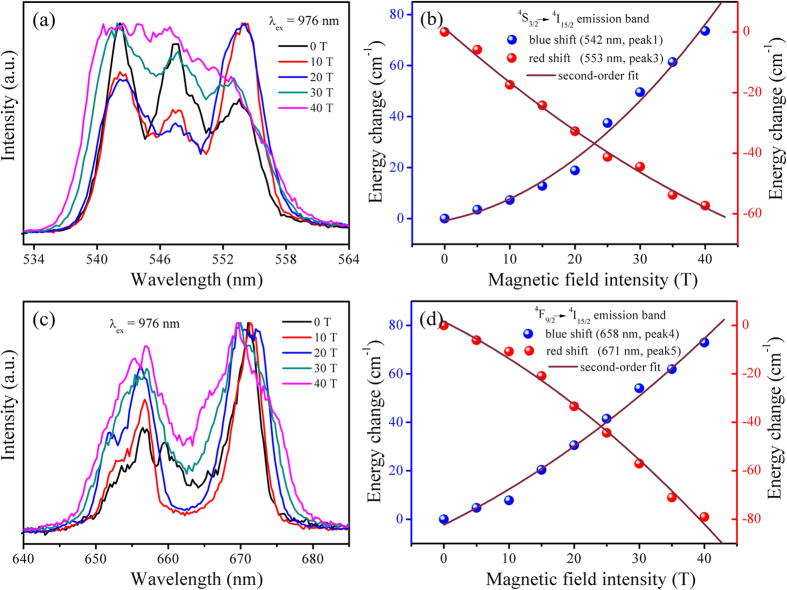
(**a**,**c**) Emission spectra of^*4*^*S*_*3/2*_ *→* ^*4*^*I*_*15/2*_ (**a**) and ^*4*^*F*_*9/2*_ *→* ^*4*^*I*_*15/2*_ (**c**) transitions of Er^3+^ ions in NaNbO_3_ nano-crystals at different magnetic fields at 77 K. (**b,d**) Dependence of energy change on the magnetic field for the transition^*4*^*S*_*3/2*_ *→* ^*4*^*I*_*15/2*_ emission band center at 611 nm (**b**) and ^*4*^*F*_*9/2*_ *→* ^*4*^*I*_*15/2*_ emission band center at 671 nm (**d**).

Apart from the transition energies and the intensity changes, it is sometimes possible to determine width of spectral lines in external magnetic fields. We will carefully study the position of emission bands of ^*4*^*S*_*3/2*_ *→* ^*4*^*I*_*15/2*_ and ^*4*^*F*_*9/2*_ *→* ^*4*^*I*_*15/2*_ transitions of Er^3+^ ions in higher magnetic field in the following. Figure 4 shows the normalized emission spectra under different magnetic fields and the dependence of energy change on the magnetic field. A blue shift at short wavelength side from 537 nm to 534 nm and red shift at long wavelength side from 557 nm to 560 nm of the emission band of Er^3+^:^*4*^*S*_*3/2*_ *→* ^*4*^*I*_*15/2*_ transition and a blue shift at short wavelength side from 650 nm to 647 nm and red shift at long wavelength side from 674 nm to 679 nm of the emission band of Er^3+^:^*4*^*F*_*9/2*_ *→* ^*4*^*I*_*15/2*_ transition can be clearly observed, which depend on the quantum nature of the respective Stark sub-levels. In the presence of the magnetic field, the external torque induced by the magnetic field adds an external energy to each energy level and increases with the strength of the magnetic field. For example, we can clearly observe that blue shift in the short wavelength edge and red shift in the long wavelength edge for Er^3+^:^*4*^*S*_*3/2*_ *→* ^*4*^*I*_*15/2*_ transition in Fig. 4(a), this is caused by the gaps among the Stark sub-levels of the ^*4*^*S*_*3/2*_ level which has a larger total momentum expand notably in the magnetic field. The magnetic field induced change of position can be described by the following equation (the related derivation shows in Supplementary)^14^,^26^:


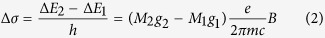


in which, Δσ is the energy change in wave-number (cm^−1^), Δ*E*_1_ and Δ*E*_2_ are the external energy before and after transition in the magnetic field, respectively.

The equation (2) indicates the energy gap between the Stark sub-levels becomes larger with the increase of magnetic field, the upper sub-bands shift to higher position in the energy level diagram while the lower sub-bands move to lower position, resulting in broadening of emission bands. For the Er^3+^:^*4*^*S*_*3/2*_ *→* ^*4*^*I*_*15/2*_ and ^*4*^*F*_*9/2*_ *→* ^*4*^*I*_*15/2*_ transitions in NaNbO_3_ nano-crystals, the relationships between the change of the emission band position and the magnetic field intensity are shown in Fig. 4(b,d). The influence of the energy change on the magnetic field intensity is nonlinear in the applied magnetic fields, which is slightly mismatch with the linear relationship according to equation (2). This nonlinear dependence could be attributed to the variation of the Lande 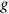 factor with the increase of magnetic field^14^,^15^. The detailed mechanism remains to be revealed in further studies.

The up-conversion emission intensity *I*_*UP*_ is proportional to the *n*th power of infrared excitation intensity *I*_*IR*_, that is:


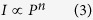


where *n* is the number of pumping photons required to excite rare earth ions from the ground state to the emitting state^27^,^28^.

**Figure 5:**
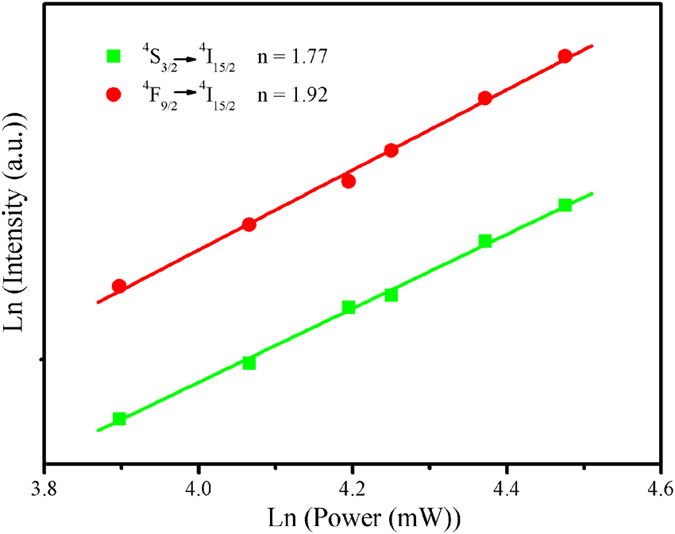
Pump power dependence of up-conversion emissions for the ^*4*^*S*_*3/2*_ *→* ^*4*^*I*_*15/2*_ and ^*4*^*F*_*9/2*_ *→* ^*4*^*I*_*15/2*_ transitions of the Er^3+^ ions (excited by 976 nm laser diode) in Er^3+^/Yb^3+^ co-doped NaNbO_3_ nano-crystals.

The dependence of up-conversion emission intensities on the pumping light intensities is measured and the ln-ln plot is drawn in Fig. 5. We find that the *n* values for the ^*4*^*S*_*3/2*_ *→* ^*4*^*I*_*15/2*_ and ^*4*^*F*_*9/2*_ *→* ^*4*^*I*_*15/2*_ emission bands are 1.77 and 1.92, respectively. It indicates that the occurrence of ^*4*^*S*_*3/2*_ *→* ^*4*^*I*_*15/2*_ emission and ^*4*^*F*_*9/2*_ *→* ^*4*^*I*_*15/2*_ emission can be attributed to a two-photon process. The two-photon process corresponds to the up-conversion emission can be described as follows: under a 976 nm laser diode excitation, the Yb^3+^ ion is excited to the ^*2*^*F*_*5/2*_ state via an incoming pump photon, and then goes back to the ground state by transferring its energy to the Er^3+^ ion in the ground state, this promotes Er^3+^ ion from the ground state ^*4*^*I*_*15/2*_ transit to the excited state ^*4*^*I*_*11/2*_. Meanwhile, some of the exited ions relax rapidly to the low-lying level of ^*4*^*I*_*13/2*_. A second 976 nm photon or energy transfer from an Yb^3+^ ion can then populate the ^*4*^*F*_*7/2*_ level of the Er^3+^ ion. Then a non-radiative decay takes place in the Er^3+^ ion through the populated state to the ^*4*^*S*_*3/2*_ level, and then the green ^*4*^*S*_*3/2*_ *→* ^*4*^*I*_*15/2*_ emissions occur. Alternatively, the ion can further relax and populate the ^*4*^*F*_*9/2*_ level, which can lead to the red ^*4*^*F*_*9/2*_ *→* ^*4*^*I*_*15/2*_ emission. The whole process of NaNbO_3_:Er^3+^/Yb^3+^ nano-crystals can be expressed as following formulas: (1) ^*2*^*F*_*7/2*_ (Yb^3+^) + 980 nm photon *→* ^*2*^*F*_*5/2*_ (Yb^3+^); (2) ^*2*^*F*_*5/2*_ (Yb^3+^) + ^*4*^*I*_*15/2*_ (Er^3+^) *→* ^*2*^*F*_*7/2*_ (Yb^3+^) + ^*4*^*I*_*11/2*_ (Er^3+^) (ET1); (3) ^*4*^*I*_*11/2*_ (Er^3+^) *→* ^*4*^*I*_*13/2*_ (Er^3+^); (4) ^*2*^*F*_*5/2*_ (Yb^3+^) + ^*4*^*I*_*13/2*_ (Er^3+^) *→* ^*2*^*F*_*7/2*_ (Yb^3+^) + ^*4*^*F*_*7/2*_ (Er^3+^) + ^*4*^*F*_*9/2*_ (Er^3+^) (ET2); (5) ^*4*^*F*_*7/2*_ (Er^3+^) *→* ^*4*^*S*_*3/2*_ (Er^3+^) + ^*4*^*F*_*9/2*_ (Er^3+^), ^*4*^*F*_*9/2*_ (Er^3+^) *→* ^*4*^*I*_*15/2*_ (Er^3+^, red); (6) ^*4*^*S*_*3/2*_ (Er^3+^) *→* ^*4*^*I*_*15/2*_ (Er^3+^, green).

**Figure 6:**
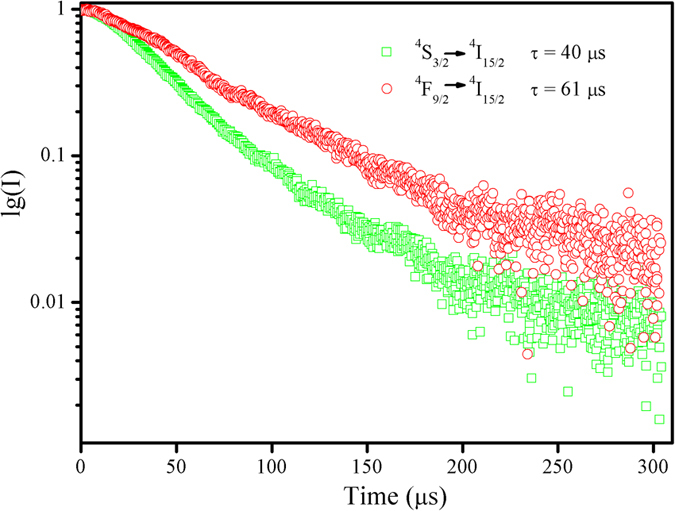
The up-conversion luminescence dynamic curves for the ^*4*^*S*_*3/2*_ *→* ^*4*^*I*_*15/2*_ and ^*4*^*F*_*9/2*_ *→* ^*4*^*I*_*15/2*_ transitions of the Er^3+^ ions (excited by 976 nm laser diode) in Er^3+^/Yb^3+^ co-doped NaNbO_3_ nano-crystals at room temperature.

To get more information about the up-conversion luminescence processes of Er^3+^ ions in NaNbO_3_:Er^3+^/Yb^3+^ nano-crystals, the decay curves of ^*4*^*S*_*3/2*_ *→* ^*4*^*I*_*15/2*_ and ^*4*^*F*_*9/2*_ *→* ^*4*^*I*_*15/2*_ transitions of Er^3+^ ions are measured under the excitation 976 nm. As shown in Fig. 6, the decay curves of Er^3+^ (λ_exc_: 976 nm) can be well fitted into a single exponential function as^29^,^30^:


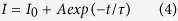


where *I* and *I*_*0*_ are the luminescence intensity at time *t* and 0, respectively, *A* is constant, *t* is the time, and *τ* represents the lifetime for the exponent. The lifetimes of ^*4*^*S*_*3/2*_ and ^*4*^*F*_*9/2*_ states of Er^3+^ ions for NaNbO_3_:Er^3+^/Yb^3+^ nano-crystals are 40 and 61 μs, respectively.

**Figure 7:**
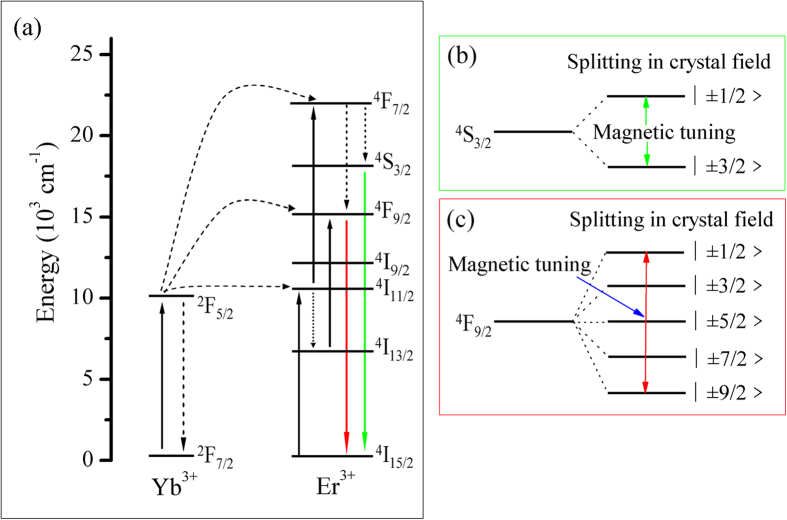
Energy levels of Er^3+^ ions (**a**) and the crystal field splitting of energy levels of ^*4*^*S*_*3/2*_ (**b**) and ^*4*^*F*_*9/2*_ (**c**) of the Er^3+^ ion in NaNbO_3_ nano-crystals.

Possible magnetic tuning luminescence processes are proposed under an external magnetic field, shown in the Fig. 7. The ^*4*^*S*_*3/2*_ *→* ^*4*^*I*_*15/2*_ emission and ^*4*^*F*_*9/2*_ *→* ^*4*^*I*_*15/2*_ emission of Er^3+^ ions in NaNbO_3_:Er^3+^/Yb^3+^ nano-crystals are both a two-photon process. The lowing of the site symmetry results in splitting of the ^*4*^*S*_*3/2*_ multiplet into two Kramers doublets, which are characterized by the projections ◽ ± 1/2> and ◽ ± 3/2> of the total momentum *J* = 3/2, and the ^*4*^*F*_*9/2*_ multiplet splits into five Kramers doublets, which are characterized by the projections ◽ ± 1/2>, ◽ ± 3/2>, ◽ ± 5/2>, ◽ ± 7/2> and ◽ ± 9/2> of the total momentum *J* = 9/2 (in which, some Stark sub-levels are crystal field degeneracy). When an external magnetic field is applied, the non-radiation transition from ^*4*^*I*_*11/2*_ to ^*4*^*I*_*13/2*_ of Er^3+^ ions is enhanced and the “mixing” effect is occurred by magnetic field, which could affect the transition energies and determine the intensity and width of spectral lines.

## Conclusions

In conclusion, magnetic-optical bi-functional NaNbO_3_:Er^3+^,Yb^3+^ nano-crystals have been successfully synthesized by the Pechini sol-gel method, consisting of a luminescent center Er^3+^ ion. Up-conversion luminescence properties of NaNbO_3_:Er^3+^,Yb^3+^ nano-crystals is efficiently tuned by applying a magnetic field at low temperature. The broadening of up-conversion spectra were observed with magnetic field, which could be ascribed to the “mixing” effect induced by magnetic field and the difference of *g* factor of sub-bands. The enhanced up-conversion luminescence with the rise of magnetic field intensity was observed, which could be mainly owing to the non-radiation transition from ^*4*^*I*_*11/2*_ to ^*4*^*I*_*13/2*_ of Er^3+^ ions is enhanced and the “mixing” effect in crystal field is occurred by an external magnetic field. This bi-functional material with controllable optical-magnetic interactions has potential applications in high accuracy communication, magnetic resonance imaging (MRI), drug targeting or carrier, aircraft guidance and optical detection of magnetic field.

## Methods

### Materials synthesis

Nano-crystals of sodium niobate (NaNbO_3_) co-doped with lanthanide (Ln = Er, Yb) (the dopants 2% Er^3+^, 20% Yb^3+^ with respect to Na^+^ ions in the structure) were successfully prepared using the Pechini sol-gel method. The starting materials were sodium carbonate (Na_2_CO_3_, A.R.), lanthanide nitrate (Ln(NO_3_)_3_·xH_2_O, 99.99%), ammonium niobium oxalate ((NH_4_)_3_[NbO(C_2_O_4_)_3_], A.R.), citric acid (CA, A.R.) and ethylene glycol (EG, A.R.). 0.1 mol% of CA was added to 10 mL of water under stirring and under heating at 90°C in a crucible. After dissolution of CA, a proper amount of (NH_4_)_3_[NbO(C_2_O_4_)_3_] was dissolved in water and then stoichiometric quantities of Na_2_CO_3_ and Ln(NO_3_)_3_·xH_2_O were added to the transparent solution and then EG was also added in the solution. The final mixtures were stirred thoroughly and heated at 120°C for 6 h until transparent brown or yellow gels were obtained. Finally, to obtain the NaNbO_3_:Er^3+^,Yb^3+^ nano-crystals, the gel precursors were calcined at 700°C for 5 h in a muffle furnace in atmosphere.

### Characterization methods

The crystal structure and phase purity of as-prepared samples were investigated by X-ray diffraction (XRD) (Bruker, D8 ADVANCE analysis with Cu K*α* radiation operated at 40 kV and 40 mA, *λ* = 0.15418 nm, scanning step 0.02°, scanning speed 0.1 s per step). The morphology and size distribution of the samples were observed by high-resolution transmission electron microscopy (HRTEM, JEOL 2100 F). The up-conversion emissions at room temperature were measured with a high-resolution spectrofluorometer (FLS920, Edinburgh Instruments, Livingston, UK) equipped with a 976 nm laser diode as excitation source. 800 nm long-pass (LP) filter (Andover, Salem, NH) was used to cut off the short wavelength lights of the simulated laser source. The up-conversion luminescence lifetimes were collected with a spectrometer (Omni-λ3007, Zolix, Beijing, China) with a digital oscilloscope (TDS 2012B, Tektronix, Beaverton, OR) and a pulse laser diode as excitation source (LE-LS-976-5000TFCA, LEO Photoelectric, Shenzhen, China).

**Figure 8:**
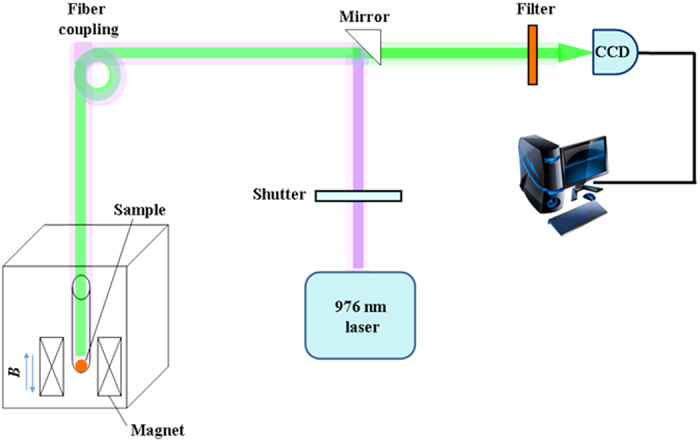
Schematic diagram for the low temperature magneto-upconversion luminescence (MUL) measurements in external pulsed magnetic fields.

The MUL spectra under pulsed magnetic field were measured using a similar fiber optical system reported previously^16^,^17^. Schematic for the MUL experiments in pulsed magnetic fields is shown in Fig. 8. The pulsed magnetic field up to 40 T was generated by a liquid nitrogen-cooled resistive coil magnet with a pulsed duration of 290 ms and the falling side of 270 ms, which was applied to the sample. The sample was placed into the center of the magnetic field through an optical probe. A laser beam irradiated by 976 nm radiation from a diode laser was launched into the probe through a multimode fiber and directly illuminated on the sample. The MUL spectrum was collected by the same fiber. The MUL signal was recorded by an EM-CCD (Andor, DU970P) through a monochromator (Andor, SR500). All measurements were investigated at room temperature, except that the measurement of MUL spectrum in magnetic field was cooled to 77 K.

## Additional Information

**How to cite this article**: Xiao, Q. *et al*. Dynamically Tuning the Up-conversion Luminescence of Er^3+^/Yb^3+^ Co-doped Sodium Niobate Nano-crystals through Magnetic Field. *Sci. Rep*. **6**, 31327; doi: 10.1038/srep31327 (2016).

## Supplementary Material

Supplementary Information
