# Supplementary material for: Dynamically Tuning the Up-conversion Luminescence of Er3+/Yb3+ Co-doped Sodium Niobate Nano-crystals through Magnetic Field
Source: Sci Rep. 2016 Aug 9;6:31327. doi: 10.1038/srep31327 (PMC4977560; doi:10.1038/srep31327)
Supplement: Supplementary Information [file srep31327-s1.doc]

**Supplementary Information**

**Dynamically Tuning the Up-conversion Luminescence of Er^3+^/Yb^3+^ Co-doped Sodium Niobate Nano-crystals through Magnetic Field**

Quan-Lan Xiao^1,2^, Yuan-Hao Zhang^2^, Han Zhang^1*^, Guo-Ping Dong^2*^, Jun-Bo Han^3^ & Jian-Rong Qiu^2*^

^1^Shenzhen Key Laboratory of Two-dimensional Materials and Devices (SKTMD), SZU-NUS Collaborative Innovation Center for Optoelectronic Science and Technology, and Key Laboratory of Optoelectronic Devices and Systems of Ministry of Education and Guangdong Province, Shenzhen University, Shenzhen 518060, P.R. China

^2^State Key Laboratory of Luminescent Materials and Devices and Institute of Optical Communication Materials, South China University of Technology, Guangzhou 510640, P.R. China

^3^Wuhan National High Magnetic Field Center, Huazhong University of Science and Technology, Wuhan 430074, P.R. China

*Correspondence to [[hzhang@szu.edu.cn](mailto:junbo.han@mail.hust.edu.cn) (H. Zhang), [dgp@scut.edu.cn](mailto:dgp@scut.edu.cn) (G. Dong) and [qjr@scut.edu.cn](mailto:qjr@scut.edu.cn) (J. Qiu)]

**The effect of magnetic field on the energy levels of rare-earth (RE) ions** ^1^

Orbital angular momentum of the electron is $P_{L}$: $P_{L}=\sqrt{L(L+1)h}$ (1)

Orbital magnetic moment is $\mu_{L}$: $\mu_{L}=\frac{e}{2mc}P_{L}$ (2)

Spin angular momentum is $P_{S}$: $P_{S}=\sqrt{S(S+1)h}$ (3)

Spin magnetic moment is $\mu_{S}$: $\mu_{S}=\frac{e}{mc}P_{S}$ (4)

in which, $e$ is the charge of an electron, $m$ is the mass of an electron, $L$ is the orbital quantum number, $S$ is the spin quantum number. $P_{J}$ is the total angular momentum, $\mu$ is the total magnetic moment. $\mu_{J}$ is the effect total magnetic moment to total angular momentum.

$\mu_{J}=g\frac{e}{2m}P_{J}$ (5)

$g$is Lande factor.

After application of magnetic field ($B$), an external torque ($L$) would be appeared, resulting in an external energy ($\Delta E$) to each energy level.

$L=\mu_{J}\times B$ (6)

$\Delta E=-\mu_{J}B\cos\beta$ (7)

$\Delta E=g\frac{e}{2m}P_{J}B\cos\beta$ (8)

$P_{J}\cos\beta=Mh$ (9)

$\Delta E=Mg\frac{eh}{2m}B$ (10)

$M$ is magnetic quantum number, which has one of 2*J*+1 values, -*J*, -*J*+1, …, *J*.

We define the energy before and after transition are $E_{1}$ and $E_{2}$, respectively, and the frequency of luminescence is $\nu$. The external energy before and after transition are $\Delta E_{1}$ and $\Delta E_{2}$ with magnetic field, respectively. The frequency of luminescence after application of magnetic field is $\nu^{'}$. The energy change in frequency is $\Delta\nu$. The energy change in wave-number is $\Delta\sigma$.

$\nu=\frac{E_{2}-E_{1}}{h}$ (11)

$\nu^{'}=\frac{E_{2}-E_{1}}{h}+\frac{\Delta E_{2}-\Delta E_{1}}{h}$ (12)

$\Delta\nu=\nu^{'}-\nu=\frac{\Delta E_{2}-\Delta E_{1}}{h}=(M_{2}g_{2}-M_{1}g_{1})\frac{e}{2\pi m}B$ (13)

$\Delta\sigma=\nu^{'}-\nu=\frac{\Delta E_{2}-\Delta E_{1}}{h}=(M_{2}g_{2}-M_{1}g_{1})\frac{e}{2\pi mc}B$ (14)

**Reference**

[1] Drake, G. W. F. In *Springer Handbook of Atomic, Molecular, and Optical Physics*, Vol. 2 (Eds: W. Martin, W. Wiese), Springer New York, New York, USA, pp.175-198(2006).
